# Zooplankton-based trophic state indices assessment of reservoirs and lakes in Central Mexico

**DOI:** 10.1007/s10661-025-14599-x

**Published:** 2025-10-06

**Authors:** Cristian Alberto Espinosa-Rodríguez, Luis Ángel Martínez-Vargas, Karla De la Luz-Vázquez, Laura Peralta-Soriano, Ligia Rivera-De la Parra, Alfonso Lugo-Vázquez

**Affiliations:** 1 Grupo de Investigación en Limnología Tropical, UIICSE, FES Iztacala, Universidad Nacional Autónoma de México, Av. De los Barrios 1, Col. Los Reyes Iztacala, Tlalnepantla, CP 54090 Estado de México Mexico; 2 Departamento de Suelos, Universidad Autónoma Chapingo, CP 56230 Texcoco, Estado de México Mexico; 3 Laboratorio de Fisiología Vegetal, L-204, FES Iztacala, Universidad Nacional Autónoma de México, Av. De los Barrios 1, Col. Los Reyes Iztacala, Tlalnepantla, CP 54090 Estado de México Mexico

**Keywords:** Index, Eutrophication, Environmental variables, Diversity, Guild ratio

## Abstract

Eutrophication presents a significant challenge for water quality management, as it threatens both the availability of freshwater and the integrity of aquatic ecosystems. Here, we integrally assessed the trophic state of ten reservoirs and lakes in Central Mexico that differ in trophic level. Environmental variables, Carlson Trophic State Index (TSI), trophic state indices for zooplankton abundances (TSI_ROT_ and TSI_CR_), and modified guild ratio (GR′) were analyzed. We also compared these indices with diversity indices (species richness, abundance, Hill numbers, and Shannon index). Sites with low Carlson TSI values exhibit reduced species richness, abundance, and diversity, while highly eutrophic and macrophyte-influenced sites display elevated diversity indicators. The modified guild ratio indicates a dominance of raptorial rotifers in reservoirs and lakes with low trophic levels, whereas microphagous species primarily dominate eutrophic ecosystems. The zooplankton indices (TSI_ROT_ and TSI_CR_) overestimate the trophic status when it is low and underestimate it when trophic conditions are high compared with Carlson’s trophic indices. Canonical correspondence analysis shows that *Asplanchna priodonta*, *Synchaeta* spp., and *Daphnia* spp. are associated with more transparent, less nutrient-rich waters, while brachionids, *Filinia longiseta*, and *Diaphanosoma* spp. thrive in eutrophic conditions like those in Xochimilco and La Estanzuela wetlands. This study confirms that zooplankton can be a good indicator of the trophic conditions of water bodies in high-altitude tropical regions, but the indices need to be adjusted to improve their accuracy.

## Introduction

Eutrophication is one of the leading causes of the deterioration of epicontinental aquatic ecosystems (Downing, 2014). This process modifies the habitat by increasing phytoplankton biomass with toxic potential, which impacts public health and causes strong fluctuations in oxygen availability (Dodds & Whiles, 2010). As a result, it threatens biodiversity and compromises the provision of essential ecosystem services (Janssen et al., 2020). Furthermore, global warming acts synergistically with eutrophication, amplifying its impacts by promoting harmful algal blooms, accelerating oxygen depletion, and placing additional strain on freshwater availability for human consumption (Moss et al., 2011; Wang et al., 2025). In response to this situation, monitoring and ecological restoration programs focused on preventing and controlling eutrophication have been implemented over the past few decades; however, they are still very costly. Therefore, research continues with the objective of achieving sustainable ecosystem management that can be widely applied (Jeppesen et al., 2012; Meerhoff et al., 2022). The integration of ecological and trophic indices into water quality monitoring would improve the environmental relevance, sensitivity, and diagnostic effectivity of lake monitoring programs (Jeppesen et al., 2011).

In 1977, Carlson proposed the Trophic State Index (TSI), which indicates anthropogenic influence on water quality and the ecological functioning of freshwater ecosystems. This index represents a quantitative scale for classifying lakes based on their nutrient status and productivity, as determined by Secchi disk transparency (SD), total phosphorus concentration, and chlorophyll *a* (Chl) content (Carlson, 1977). Some studies have discussed the unsuitability of Carlson TSI for tropical and subtropical ecosystems (Cunha et al., 2013, 2021; Klippel et al., 2020); nonetheless, there are no modifications for high-altitude tropical ecosystems where temperature attenuation represents a key variable influencing weather and stratification (MacIntyre & Hamilton, 2023). On the other hand, biological indicators and indices for monitoring the trophic state of reservoirs and lakes using organisms offer a practical and economical tool for water quality assessment (Chandel et al., 2024; Michaloudi et al., 2024).

One of the primary components in monitoring and restoring lakes and reservoirs is metazooplankton, which comprises rotifers, cladocerans, and copepods (Jeppesen et al., 2011). They have proven effective as bioindicators of water quality (Jeppesen et al., 2007, 2012), paleoclimatic, and paleoenvironmental conditions (Wojewódka et al., 2020), as well as trophic status in temperate (Duggan et al., 2001a; Ejsmont-Karabin, 2012; Ejsmont-Karabin & Karabin, 2013) and tropical environments (Gazonato et al., 2014; Perbiche-Neves et al., 2016). They occupy an intermediate trophic position as consumers of phytoplankton and food for macroinvertebrates and fish, and they respond quickly to changing environmental conditions. They have short generation times and high reproductive rates (Beisner & Thackeray, 2023). These characteristics make zooplankton an excellent tool for detecting changes in environmental conditions, which can be produced by a wide range of stressors such as increased temperature, pollution, and invasive species (Michaloudi et al., 2024).

Using zooplankton as a tool for trophic state assessment is well-established in ecological research (Ejsmont-Karabin, 2012; Ejsmont-Karabin & Karabin, 2013; Jeppesen et al., 2011; Montagud et al., 2019). The saprobic index and the *Brachionus*/*Trichocerca* quotient, where taxonomical identification was crucial, were the first attempts to use zooplankton as indicators of environmental quality (Slácedêcek, 1983). More recently, indices based on biomass and abundance of rotifers (TSI_ROT_) and crustaceans (TSI_CR_) were proposed (Ejsmont-Karabin, 2012; Ejsmont-Karabin & Karabin, 2013) and tested for different conditions with contrasting results (Kuczyńska-Kippen et al., 2025; Stamou et al., 2019). Furthermore, the trait-based index guild ratio for rotifers, considering the proportion of raptorial to microphagous species, reflects the influence of environmental conditions such as eutrophication (Obertegger et al., 2011).

In Central Mexico, the country’s wide diversity of ecosystems and species is closely linked to its geographical location (Alcocer et al., 2022). Altitude is a key factor in aquatic system studies, as variations in physical and chemical characteristics influence biological composition (Alcocer & Bernal-Brooks, 2010). High-altitude systems are especially valuable as natural laboratories for understanding how species diversity changes in tropical regions with specific environmental conditions (Banderas et al., 1991). These systems also hold significant cultural and economic value and host diverse native species, including many zooplankton taxa (Chacón & Rosas-Monge, 1998), which are increasingly impacted by environmental degradation.

Several studies have analyzed the annual dynamics of metazooplankton in different lakes and reservoirs in Central Mexico (Ciros-Pérez et al., 2015; Espinosa-Rodríguez et al., 2021; García-García et al., 2012), but there is no regional analysis that helps us to establish the determining factors in the structuring of the zooplankton community, nor an analysis of zooplankton ecological indicators that considers water bodies with diverse environmental conditions (Alcocer et al., 2022). This study aimed to assess the trophic state of aquatic systems in Central Mexico by integrating limnological variables, Carlson’s Trophic State Index (TSI_SD_ and TSI_CHL_), zooplankton abundance-based trophic indices (TSI_ROT_ and TSI_CR_), zooplankton diversity, and the modified guild ratio (GR′). We also compare the effectiveness of these indices in adequately expressing the trophic status of aquatic systems located in high-altitude tropical regions.

## Materials and methods

### Study area

**Fig. 1:**
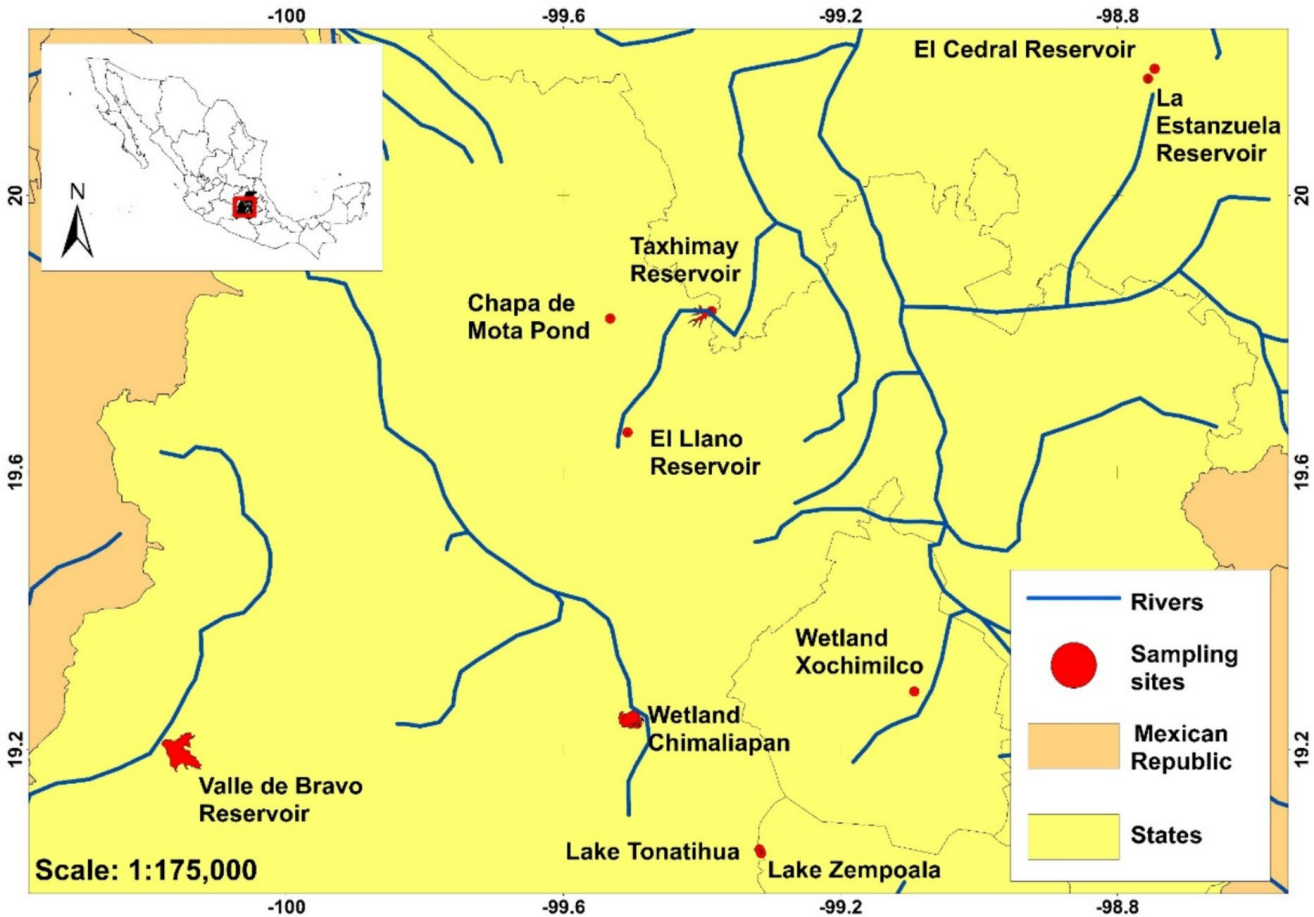
Location of the study area in Central Mexico. Red dots represent the 10 aquatic systems and sampling sites

The study area (Fig. 1) is located between coordinates 19° 03′ 01″, 20° 10′ 55″ N and 99° 05′ 35″, 100° 08′ 36″ W in Central Mexico on the Mexican Volcanic Belt. Most of these lakes and reservoirs are shallow, polymictic, and have trophic levels that vary from mesotrophic to hypertrophic at altitudes higher than 1800 m a.s.l. in the Balsas, Pánuco, and Lerma-Santiago basins (Table 1), where a temperate climate with coniferous and mixed forests predominates (Alcocer & Bernal-Brooks, 2010).

**Table 1:** List of the selected sites, type of system, geographic location, altitude, max depth (Z_MAX_), area, and hydrological basin

Sampling sites	Type of system	Coordinates	Altitude (m a.s.l.)	Z_MAX_	Area	Hydrological basin
**Taxhimay (Tax)**	Reservoir	19° 49′ 55″ N 99° 23′ 55″ W	2200	25 m	365 ha	Pánuco
**La Estanzuela (Est)**	Reservoir	20° 10′ 07″ N 98° 45′ 20″ W	2756	10 m	20 ha	Pánuco
**El Cedral (Ced)**	Reservoir	20° 10′ 58″ N 98° 44′ 46″ W	2792	6 m	4 ha	Pánuco
**El Llano (Llan)**	Reservoir	19° 39′ 29″ N 99° 30′ 26″ W	2800	22 m	4.5 ha	Pánuco
**Valle de Bravo (VDB)**	Reservoir	19° 11′ 12″ N 100° 08′ 36″ W	1830	38 m	1855 ha	Balsas
**Tonatihua (Ton)**	Lake	19° 03′ 19″ N 99° 18′ 59″ W	2810	5 m	4 ha	Lerma-Santiago
**Zempoala (Zem)**	Lake	19° 03′ 01″ N 99° 18′ 50″ W	2800	7 m	11 ha	Lerma-Santiago
**Xochimilco (Xoch)**	Wetland	19° 17′ 02″ N 99° 05′ 35″ W	2240	2 m	215 ha	Valle de México
**Chimaliapan (Chim)**	Wetland	19° 14′ 06″ N 99° 29′ 14″ W	2574	5 m	675 ha	Lerma-Santiago
**Chapa de Mota (CDM)**	Pond	19° 49′ 20″ N 99° 31′ 57″ W	2585	3 m	3 ha	Pánuco

### Limnological sampling

Each water body was sampled in triplicate on the same date at the same sampling station. Aquatic systems were sampled between March and April during the dry season of 2022. We focused on the dry season to compare ecological and trophic indices among different water bodies under similar climatic conditions. In the rainy season, runoff carries large amounts of clay, reducing Secchi disk transparency in ways unrelated to productivity and causing the TSI_SD_ to overclassify the trophic state. While seasonality is essential for more accurate assessments, this study serves as a starting point for developing a comprehensive monitoring program. At each sampling point, in the upper 30 cm of the water column, temperature, dissolved oxygen (DO), and its saturation, and conductivity were measured using a YSI85 probe. At the same time, pH was recorded with a Conductronic PC-20 pH meter. Water depth was determined using a Hondex digital echosounder, and transparency was assessed using a Secchi disk. Water samples for soluble reactive phosphorus (SRP) and nitrates (NO_3_) quantification were kept at 4 °C up to 24 h before being analyzed in the laboratory using a HACH DR3900 spectrophotometer with standardized methods (APHA, 2012). Chlorophyll *a* was measured using the 90% acetone extraction and analyzed with a 10-AU Turner Designs fluorometer (Arar & Collins, 1997). To ensure quality control, triplicate zooplankton samples were examined. Water column integrated zooplankton samples were collected with a Wisconsin plankton net (64-µm mesh size) using vertical hauls in the pelagic zone, stopping 2 m above the bottom to avoid benthic contamination. For water bodies shallower than 3 m (Xochimilco and Chapa de Mota), we filtered 60 L of surface water using a bucket. The samples were preserved in formaldehyde at a final concentration of 4%.

### Zooplankton sample analysis

Triplicate zooplankton samples per site were examined under a Zeiss Axioscope A1 microscope using a Sedgwick-Rafter counting chamber, analyzing a minimum of five subsamples or 400 individuals of the most abundant species (Wetzel & Likens, 2001). Taxonomical identification was conducted at least at the genus level for rotifers and cladocerans using the keys of Koste (1978), Elías-Gutiérrez et al. (2008), Sarma and Nandini (2017), and Bielańska-Grajner et al. (2016). For copepods, we grouped them into nauplii, copepodites, and adults, and separated cyclopoid, calanoid, and harpacticoid copepods.

### Trophic state index

#### Carlson index

The trophic state of each lake and reservoir was calculated using the classical formulas proposed by Carlson (1977), considering the Secchi disk transparency and chlorophyll *a* indices according to the following Eqs. (1 and 2):

1 $${\text{TSI}}_{\text{SD}}=60-14.41\text{ln}(\text{SD})$$

2 $${\text{TSI}}_{\text{CHL}}=9.81\text{ln}(\text{CHL}a)+30.46$$

Carlson TSI assesses trophic status on a scale from 0 to 100, with each value corresponding to a specific trophic condition. Therefore, 0 < TSI ≤ 40 oligotrophic, 40 > TSI ≤ 50 mesotrophic, 50 > TSI ≤ 70 eutrophic, and 70 > TSI ≤ 100 hypereutrophic.

#### Rotifer and crustacean trophic state index

We also considered trophic state indices derived from rotifers (Ejsmont-Karabin, 2012) and crustacean abundances (Ejsmont-Karabin & Karabin, 2013) for polymictic lakes (POL) and indistinct mixis patterns (MIX), according to the following Eqs. (3–7):

3 $${\text{TSI}}_{\text{ROT}}\text{POL}(\text{rotifer abundance})=4.64\text{ ln }(N)+25.36$$

4 $${\text{TSI}}_{\text{ROT}}\text{MIX}(\text{rotifer abundance})=5.38\text{ln }(N)+19.28$$

5 $${\text{TSI}}_{\text{ROT}}\text{indicative of high trophy}(\text{IHT})=0.200\text{IHT}+41$$

6 $${\text{TSI}}_{\text{CR}1}\text{POL}(\text{crustacean abundance})=6.89\text{ln}(N)+20.7$$

7 $${\text{TSI}}_{\text{CR}1}\text{MIX }(\text{crustacean abundance})=25.5N0.142$$

These indices assess trophic state based on abundance of rotifers (TSI_ROT_) and crustaceans (TSI_CR_) as well as the percentage of indicating high trophic (IHT) rotifers such as *Brachionus* spp., *Filinia longiseta*, *Keratella cochlearis*, and *Pompholyx sulcata*. Lakes with TSI_ROT_ and TSI_CR_ values below 45 are classified as mesotrophic, between 45 and 55 as meso-eutrophic, 55 and 65 as eutrophic, and above 65 as hypertrophic (Ejsmont-Karabin, 2012).

### Modified guild ratio (GR′)

Rotifer species were classified according to the feeding strategies employed by each genus. Therefore, genera with cardate, forcipate, incudate, uncinate, and virgate trophi were grouped as raptorial, while species with malleoramate, maleate, and ramate trophi were grouped as microphagous (Obertegger et al., 2011). Initially, this index was based on rotifer biomass; however, Wen et al. (2017) compared biomass and abundance and found similar results. Thus, in this study, we used abundances of raptorial and microphagous rotifers to assess how guild ratio changes concerning different trophic conditions in high-altitude tropical lakes and reservoirs using the following Eq. (8):

8 $$G{R}^{\prime}=\left(R-M\right)/R+M$$

*R* = total abundance of raptorial individuals and *M* = total abundance of microphagous individuals. A GR′ value below 0 means the dominance of microphagous species, while a value above 0 indicates the predominance of raptorial species.

### Zooplankton community and ecological index analysis

The diversity indices were calculated from species richness (S), abundance, Shannon index (H′), and the effective number of species using Hill’s numbers (H1 and H2) (Hill, 1973; Magurran, 2004) employing the software PRIMER 7.0.24. We also classified the species as dominant, constant, occasional, and rare based on abundance and frequency with the Olmstead-Tukey corner test (Sokal & Rohlf, 1981).

To examine the relationship between temperature, conductivity, oxygen saturation, pH, Secchi transparency, depth, nitrates, SRP, and chlorophyll *a* variables and zooplankton community structure, we conducted a canonical correspondence analysis (CCA) using CANOCO 4.5 (Ter Braak & Smilauer, 1998). Environmental variables were normalized (the mean and standard deviation of each variable are calculated, and then the mean is subtracted from each variable’s data and divided by the standard deviation) to have a common scale. Zooplankton abundances were log(*x* + 1). Forward selection and the Monte Carlo permutation test were applied to assess the statistical significance of the relationships between environmental variables and species data. A multiple linear regression analysis was used to test the relationship between the values of the different zooplankton abundance trophic state indices with Carlson’s TSI_SD_ and TSI_CHL_.

## Results

### Environmental characterization

The shallowest sampling point was 0.8 ± 0.6 m in Xochimilco, and the deepest was 18.3 m in Valle de Bravo. The water temperature ranged between a minimum of 12.1 ± 0.2 °C in Reservoir La Estanzuela to a maximum of 23.9 ± 0.4 °C in Valle de Bravo. The conductivity varied from 76 ± 0.7 µS cm^−1^ in Reservoir El Llano to 737 ± 6 µS cm^−1^ in wetland Xochimilco. Oxygen saturation fluctuates from 12.4 ± 3% (1.08 mg L^−1^) in Chimaliapan Wetland to 119 ± 5% (10.04 mg L^−1^) in Xochimilco Wetland. pH values were between 7 ± 0.1 and 9.2 ± 0.1. The Secchi disk oscillated from 0.2 ± 0.1 m in Xochimilco Wetland to 4.1 ± 0.4 m in Reservoir El Cedral. For N–NO_3_^+1^, the results ranged from 0.39 mg L^−1^ in Reservoir El Cedral to 1.43 ± 0.2 mg L^−1^ in Reservoir La Estanzuela. Soluble reactive phosphorus (SRP) fluctuated between 0.01 mg L^−1^ in Chapa de Mota and Valle de Bravo to 0.38 mg L^−1^ in Reservoir La Estanzuela. Chlorophyll *a* varied from 1.3 ± 0.4 µg L^−1^ in Reservoir El Cedral to 120 ± 8 µg L^−1^ in Xochimilco Wetland (Table 2).

**Table 2:** Surface environmental variables (mean ± standard deviation) registered in aquatic systems of Central Mexico during the dry season of 2022. For abbreviation systems, see Table 1

	Tax	Est	Ced	Llan	VDB	Ton	Zem	Xoch	Chim	CDM
Temp (°C)	21.6 ± 0.4	12.1 ± 0.2	17.2 ± 0.2	14.2 ± 0.5	23.9 ± 0.4	17.1 ± 0.5	16.8 ± 0.1	23.4 ± 0.1	20.5 ± 0.1	18.4 ± 0.1
Conduc. (µS.cm^−1^)	136 ± 0.5	266 ± 1	102 ± 3	76 ± 0.7	159 ± 20	165 ± 0.5	104 ± 0.2	737 ± 6	476 ± 5	195 ± 0.5
% DO	89 ± 1	74 ± 7	79 ± 6	83 ± 1	102 ± 6	63 ± 3	74 ± 1	119 ± 5	12.4 ± 3	70 ± 0.8
DO (mg L^−1^)	7.8 ± 0.1	6.0 ± 0.5	7.6 ± 0.4	8.5 ± 0.1	8.0 ± 0.2	6.9 ± 0.2	7.1 ± 0.1	10.0 ± 0.3	1.0 ± 0.2	6.3 ± 0.1
pH	7 ± 0.1	9.2 ± 0.1	8.8 ± 0.2	7.6 ± 0.3	8.6 ± 0.1	7.7 ± 0.1	7.4 ± 0.1	7.2 ± 0.1	6.6 ± 0.1	7 ± 0.1
Depth (m)	10.8 ± 0.1	2.3 ± 0.1	6 ± 0.4	15.6 ± 0.8	18.3 ± 2.3	5 ± 0.1	6.3 ± 0.1	0.8 ± 0.6	2.3 ± 0.1	0.3 ± 0.1
Secchi (m)	1 ± 0.1	0.3 ± 0.1	4.1 ± 0.4	3.4 ± 0.8	1.2 ± 0.2	2.8 ± 0.1	3.2 ± 0.1	0.2 ± 0.1	1.8 ± 0	0.5 ± 0
N–NO_3_ (mg L^−1^)	0.92 ± 0.1	1.43 ± 0.2	0.39 ± 0	0.49 ± 0	0.4 ± 0.1	0.93 ± 0	1.14 ± 0	2.9 ± 0	0.93 ± 0.1	0.63 ± 0.1
P-SRP (mg L^−1^)	0.07 ± 0	0.38 ± 0	0.03 ± 0	0.09 ± 0	0.01 ± 0	0.06 ± 0	0.03 ± 0	2.4 ± 0	0.3 ± 0	0.01 ± 0
Chlo *a* (µg L^−1^)	32 ± 1.8	30 ± 3.7	1.3 ± 0.4	5.6 ± 2.2	25 ± 1.6	4 ± 1.1	3.5 ± 1	120 ± 8	2.7 ± 0.1	13.7 ± 2.4

### Zooplankton diversity

**Fig. 2:**
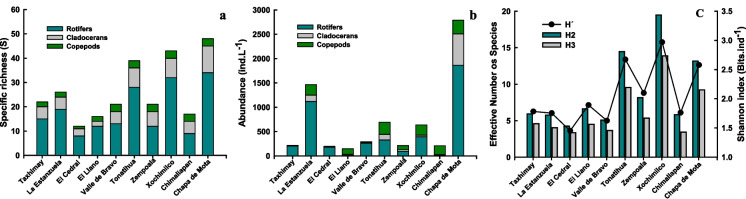
**a** Zooplankton specific richness, **b** abundance, **c** effective number of species, and Shannon diversity index in aquatic systems of Central Mexico during the dry season in 2022

Chapa de Mota (48 spp.), Xochimilco (43 spp.), and Tonatihua (39 spp.) showed the highest zooplankton species richness (Fig. 2a). In contrast, El Cedral (12 spp.), El Llano (16 spp.), and Chimaliapan (17 spp.) had the lower values. For zooplankton groups, we found eight species of rotifers in El Cedral and 34 in Chapa de Mota. In Chimaliapan, El Llano, Valle de Bravo, and Zempoala, we registered between 9 and 13 species, while in Xochimilco and Tonatihua, 32 and 28 rotifer species were counted. For cladocerans, the lowest values were recorded for El Llano and El Cedral, with 2 and 3 species, respectively. The highest number of cladocerans was found in Chapa de Mota, with 11 species. Between 5 and 8 species were registered for most waterbodies. No more than three species of copepods were recorded in the sampling sites (Fig. 2a). Maximum zooplankton abundance was recorded for Chapa de Mota and La Estanzuela, with 2621 ind L^−1^ and 1463 ind L^−1^, respectively. Minimum densities were registered in El Llano, El Cedral, Taxhimay, Zempoala, and Chimaliapan, with around 200 ind L^−1^ (Fig. 2b). The ratio of rotifers/crustaceans was higher for El Cedral (12.02), Taxhimay (10.54), and Valle de Bravo (9.25), while the lowest ratio was found in Chimaliapan (0.09), El Llano (0.20), Zempoala (0.84), and Tonatihua (0.92). For crustaceans, the cladocerans/copepods ratio was higher in El Cedral (2.59), Chapa de Mota (2.39), and Valle de Bravo (1.58), while lower values were recorded in El Llano (0.078), Chimaliapan (0.088), and Xochimilco (0.148). In Fig. 2c, diversity values are presented as the effective number of species and the Shannon index. The Shannon index had its highest value in Xochimilco (2.69 nats ind^−1^) and lowest (0.70 nats ind^−1^) in Chimaliapan. The effective number of species (H1, the Hill number of order 1), which weights the species according to their relative abundance, also places Xochimilco as the water body with the highest diversity (14.85 species) and Chimaliapan with the lowest (2.03 species). The second-order Hill number (H2) showed a similar result (Xochimilco 9.03 and Chimaliapan 1.43 effective species, respectively).

### Trophic state indices

Considering the Carlson index (Secchi disk and Chlo *a* content), TSI_SD_ classified El Cedral as oligotrophic; El Llano, Tonatihua, and Zempoala as mesotrophic; Taxhimay, Valle de Bravo, Chimaliapan, and Chapa de Mota as eutrophic; and the hypertrophic were La Estanzuela and Xochimilco. TSI_CHL_ group waterbodies in a similar way, only La Estanzuela and Chimaliapan showed discrepancies. For TSI_ROT_, based on abundances, calculations resulted in similar trophic state categories grouping most waterbodies as meso-eutrophic. Different categories were found between calculations for polymictic and indistinct mix patterns for La Estanzuela, El Llano, and Zempoala. TSI_ROT_ IHT also classified most reservoirs and lakes as meso-eutrophic, Zempoala and Tonatihua as mesotrophic, and La Estanzuela as Eutrophic. TSI_CR_, based on abundances, showed similar classification for most aquatic systems independently of the equation related to the lake mixing pattern; differences were only registered for Valle de Bravo and Chimaliapan. These indices classify Taxhimay and El Cedral as mesotrophic; El Llano, Zempoala, and Chimaliapan as meso-eutrophic; and La Estanzuela, Valle de Bravo, Tonatihua, and Xochimilco as eutrophic, and Chapa de Mota as hypertrophic (Table 3).

**Table 3:**
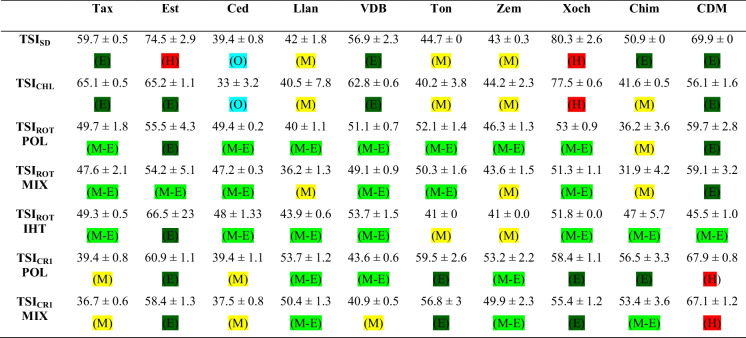
Trophic state indices for reservoirs and lakes in Central Mexico during the dry season of 2022

The multiple regression linear analysis found a significant relationship between TSI_SD_ and TSI_ROT_POL (*p* < 0.05), TSI_ROT_MIX (*p* < 0.05), TSI_CRI_POL (*p* < 0.001), and TSI_CRI_MIX (*p* < 0.001). For TSI_CHL_, the significant relationships were with TSI_CRI_POL (*p* < 0.001) and TSI_CRI_MIX (*p* < 0.001) (Table 4).

**Table 4:** Multiple lineal regression (*n* = 30) between TSI_SD_, TSI_CHL_, and the TSI of rotifers (TSI_ROT_) and crustaceans (TSI_cr1_) abundances. ns = non-significant

	TSI_ROT_POL	TSI_ROT_MIX	TSI_ROT_IHT	TSI_CR1_POL	TSI_CR1_MIX
TSI_SD_	*r*^2^ = 0.351, *p* < 0.05	*r*^2^ = 0.351, *p* < 0.05	ns	*r*^2^ = 0.48 *p* < 0.001	*r*^2^ = 0.52 *p* < 0.001
TSI_CHL_	ns	ns	ns	*r*^2^ = 0.48 *p* < 0.001	*r*^2^ = 0.52 *p* < 0.001

**Fig. 3:**
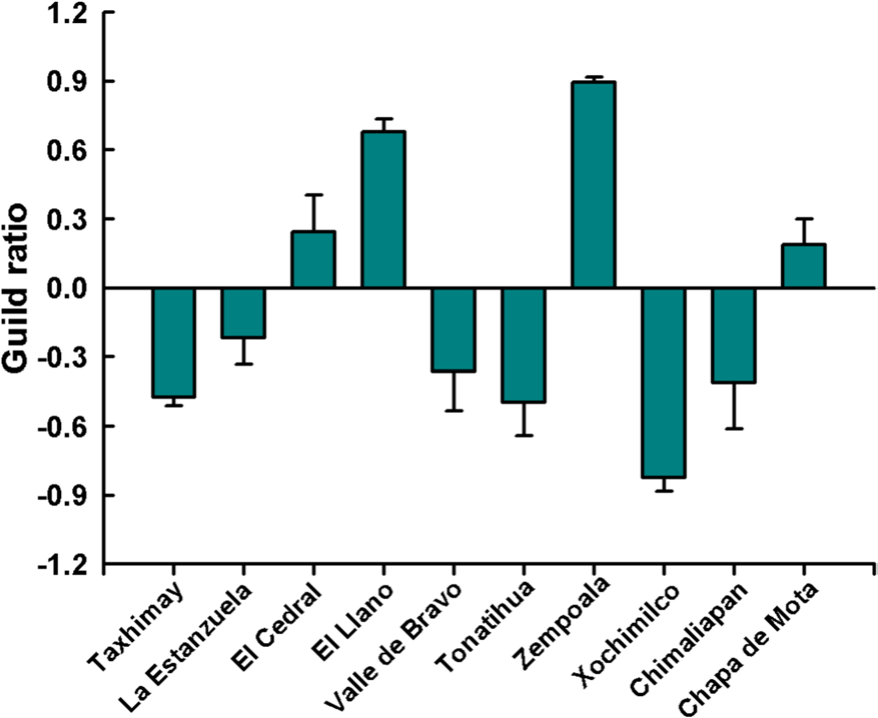
Guild ratio of rotifers from reservoirs and lakes of Central Mexico during the dry season in 2022

In El Cedral, El Llano, Zempoala, and Chapa de Mota, rotifers guild ratio values have positive values between 0.18 ± 0.11 in Chapa de Mota and 0.89 ± 0.02 in Zempoala, meaning a dominance of raptorial rotifers species. In La Estanzuela, Valle de Bravo, Chimaliapan, Taxhimay, Tonatihua, and Xochimilco, a dominance of microphagous rotifers was registered with values between − 0.21 ± 0.11 and − 0.82 ± 0.06 (Fig. 3).

### Zooplankton community analysis

**Fig. 4:**
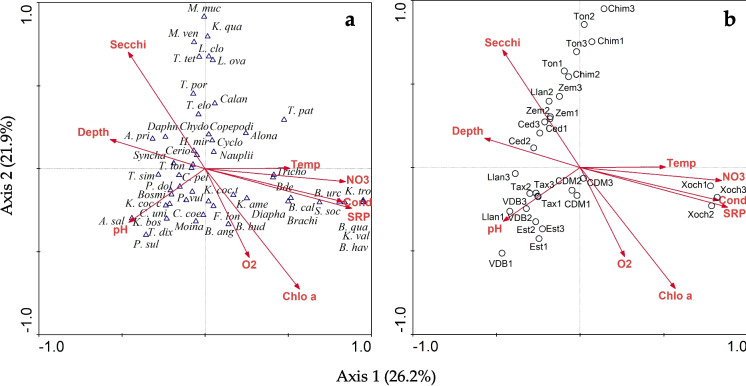
Canonical correspondence analysis of environmental variables and zooplankton (**a**) and environmental variables and reservoirs and lakes of Central Mexico (**b**) in the dry season of 2022. See code for sites on Table 1. 1–3 replications

CCA explained 48.1% of the data variation, where the variables SRP (*p* < 0.001; *F* = 5.84; *λ* = 0.35) and chlorophyll *a* (*p* < 0.001; *F* = 4.98; *λ* = 0.26) were statistically significant. These variables were positively related to nitrates and conductivity and negatively associated with the Secchi disk and depth. *Brachionus* spp., *Keratella* spp., *Filinia longiseta*, bdelloids, and *Diaphanosoma* sp. were positively associated with an increase in nutrients, chlorophyll *a*, and conductivity, indicating a preference for eutrophic conditions. In contrast, *Asplanchna priodonta*, *Daphnia* spp., and *Synchaeta* spp. were negatively associated with these variables and positively associated with greater depth and water transparency (Fig. 4a). In the triplots with aquatic systems (Fig. 4b), reservoirs El Cedral and El Llano as well as lakes Zempoala and Tonatihua, were associated with a decrease in chlorophyll *a*, nutrients, and conductivity, and positively related to Secchi disk depth. In the other extreme, the wetland Xochimilco shows a positive relation with an increase of conductivity, nitrates, SRP, and chlorophyll *a* (Fig. 4b).

## Discussion

### Environmental variation

The Mexican Volcanic Belt is considered a high-altitude region where the climate resembles that of temperate latitudes. The temperature of the aquatic systems in this area ranges from 12 to 25 °C (Alcocer & Bernal-Brooks, 2010; Espinosa-Rodríguez et al., 2021; Jiménez-Santos et al., 2019), similar to those registered in this study. The geology of the volcanic range around the Mexico basin, where most of the reservoirs and lakes studied here are located, is mainly composed of volcanic rock such as andesite and dacite with relatively low solubility (Arce et al., 2019), resulting in low mineralized reservoirs and lakes. Oxygen was oversaturated in Valle de Bravo and Xochimilco, which have a eutrophic condition where diurnal oxygen concentration strongly fluctuates due to photosynthesis processes (Dodds & Whiles, 2010). In Chimaliapan, the low oxygen concentration is related to high respiration rates because the watercolor was brownish, and this condition has been related to dystrophy and oxygen reduction (Sepp et al., 2018).

### Trophic state indices

The trophic state analysis is crucial for developing strategies for eutrophication control and lake management (Carlson, 1977; Cunha et al., 2013). Table 5 compares previous trophic state records for reservoirs and lakes in Central Mexico with the results of this study. Due to the pronounced seasonality of the region, our estimates collected during a low-productivity period may underestimate the actual trophic state, highlighting the need for further research across seasons. Although the TSI_SD_ has been criticized for its susceptibility to interference from particulate inorganic matter, such as clay and silt, which reduce light penetration (Alcocer & Bernal-Brooks, 2010), this effect is less pronounced during the dry season, when the absence of rainfall minimizes non-biogenic turbidity from surface runoff. Overall, our findings are broadly consistent with earlier studies employing different trophic state assessment methods.

**Table 5:** Comparison of trophic state determinations between this study and previous studies in aquatic systems of Central Mexico

	Present TSI	Previous TSI	TSI equation	References
Tax	Meso-eutrophic	Eutrophic	Association quotient; IP of Palmer; diatom index	Garduño-Solorzano et al. 2025
Est	Eutrophic	None		
Ced	Oligo-mesotrophic	None		
Llan	Mesotrophic	Oligotrophic, mesotrophic, eutrophic	Saprobic index; TSI_ROT_; *B*:*T* quotient; association quotient; IP of Palmer; diatom index	González-Gutiérrez et al., 2023; Garduño-Solorzano et al. 2025
VDB	Meso-eutrophic	Meso-eutrophic, Eutrophic	Carlson TSI; chlorophyll *a* concentration	Merino-Ibarra et al., 2008; Bravo-Inclán et al., 2011
Ton	Meso-eutrophic	None		
Zem	Mesotrophic	Mesotrophic	Chlorophyll *a* concentration	Sigala et al., 2017
Xoch	Eu-hypertrophic	Hypertrophic	Carlson TSI; TSI_ROT_	Jiménez-Santos et al., 2019
Chim	Meso-eutrophic	Meso-eutrophic	*B*:*T* quotient	García-García et al., 2012
CDM	Eu-hypertrophic	None		

TSI_SD_ and TSI_CHL_ were consistent in most cases, showing discrepancies only for Chimaliapan and La Estanzuela; in both cases, chlorophyll *a* underestimated the trophic state compared to the Secchi disk. Phytoplankton is considered the most suitable group for trophic state estimation due to its short generation time and rapid response to fluctuations in nutrient availability (Stamou et al., 2019). Likewise, chlorophyll *a* has been widely used as a proxy for phytoplankton biomass (Jeppesen et al., 2011). Nevertheless, some studies have shown that chlorophyll *a* can be a poor predictor of trophic state because it strongly depends on algal composition and physiological condition (Cunha et al., 2021; Stamou et al., 2019). This limitation is particularly relevant in tropical and subtropical ecosystems, where some studies suggest that the Carlson TSI is not suitable (Cunha et al., 2013; Klippel et al., 2020). However, given the absence of indices specifically developed for high-altitude tropical systems, we used it here as a reference point.

Some studies conducted in temperate and tropical regions have shown a weak correlation between total phosphorus and chlorophyll *a*, suggesting that phosphorus may not be the primary limiting factor for phytoplankton abundance (Karpowicz et al., 2025; Klippel et al., 2020); for this reason, total phosphorus was not considered in determining the TSI_TP_ (Carlson, 1977). Moreover, studies in Central Mexico indicate that reservoirs and lakes have relatively high total phosphorus and nitrogen concentrations, with the former being the limiting nutrient for primary productivity (Alcocer & Bernal-Brooks, 2010; Arce et al., 2019).

The use of zooplankton as an indicator of the trophic status of inland water bodies has been proposed by various authors (Slácedêcek, 1983; Duggan et al., 2001a; Jeppesen et al., 2011; Ejsmont-Karabin, 2012; Ejsmont-Karabin & Karabin, 2013 Gazonato et al., 2014; Perbiche-Neves, 2016; Montagud et al., 2019; Stamou et al., 2019; Chandel et al., 2024; Michaloudi et al., 2024; Kuczyńska-Kippen et al., 2025). One advantage of using TSI_ROT_, TSI_CR_, and guild ratio is that taxonomical determination to species level is not necessary (Ejsmont-Karabin, 2012; Ejsmont-Karabin & Karabin, 2013; Obertegger et al., 2011). The most comprehensive proposal is that of Ejsmont-Karabin (2012), who proposed various trophic indices based on the abundance of rotifers. Ejsmont-Karabin and Karabin (2013) also developed trophic indices based on microcrustacean abundances. However, several of these authors agree that each water body has its particularities, making it difficult to develop general indices. Inconsistencies between Carlson and zooplankton indices found in this study may also be related to zooplankton seasonality. In some lakes and reservoirs of Central Mexico, rotifers are abundant during the rainy season, when productivity increases, but their abundance declines during the dry season (Espinosa-Rodríguez et al., 2021; García-García et al., 2012). Nevertheless, less information is available for crustacean zooplankton. Thus, comparing seasonal changes represents an important opportunity for further research on this topic. Furthermore, trophic state misclassifications have been reported along the eutrophication gradient, highlighting the importance of developing indices adjusted to specific environmental conditions (Cunha et al., 2013; Montagud et al., 2019; Stamou et al., 2019).

Rotifers are highly responsive to environmental disturbances, making them effective indicators of pollution and changes in trophic state (Ejsmont-Karabin, 2012; Slácedêcek, 1983). Current approaches highlight the importance of testing functional-based variables (Wen et al., 2017). The guild ratio is associated with the trophic level of reservoirs and lakes in Central Mexico, where the dominance of microphagous rotifers is higher in eutrophic and hypertrophic Xochimilco, La Estanzuela, Taxhimay, Valle de Bravo, and Chimaliapan (Ejsmont-Karabin, 2012), while the dominance of raptorial rotifers is related to oligotrophic and mesotrophic systems such as El Llano, El Cedral, and Zempoala. The dominance of microphagous rotifers coincides with a low abundance of cladocerans in Taxhimay, Valle de Bravo, Xochimilco, and Chimaliapan, and a similar trend has been reported for Lakes Caldonazzo and Washington (Obertegger et al., 2011). For Tonatihua and Chimaliapan, the guild ratio did not align with the Carlson TSI, likely due to the presence of high macrophyte biomass and the shallow depth at the sampling sites. Wallace et al. (2021) found that benthic rotifers (sometimes also associated with macrophytes) do not show differences in trophic type across trophic states but do exhibit differences at the species composition level, which is consistent with the high species richness, Shannon index, and Hill numbers found in these water bodies. Duggan et al. (2001b) reported that rotifer abundance strongly depends on macrophyte species and biomass within the same ecosystem; therefore, in vegetated areas, TSI based on abundances would reflect macrophyte influence more than trophic level. Consequently, littoral areas remain an important opportunity for research on how zooplankton communities vary in relation to trophic state.

Overall, TSI_ROT_ and TSI_CR_ overestimate values at low trophic levels compared to the Carlson indices and underestimate values at high trophic levels. However, the level of underestimation and overestimation is greater with rotifers. In the lineal regression analysis, the TSI_SD_ had a greater number of significant relationships (4) with the biological TSIs, but the *r*^2^ values were higher with the crustacean-based indices. The TSI_CHL_ was only related to the crustacean-based indices. The relationship between water transparency and biological indices may have been favored by the dry season, when rainfall does not carry significant amounts of suspended solids into the water bodies, which affect the measurement of transparency but do not contribute to primary production. Trophic state misclassifications have been reported along the eutrophication gradient, highlighting the importance of developing indices adjusted to specific environmental conditions (Stamou et al., 2019) and temporal variations.

### Zooplankton community and trophic state

The dominant rotifers in our study area were *K. cochlearis*, *Polyarthra vulgaris*, *P. dolichoptera*, *Synchaeta* sp., and *Trichocerca similis*, while crustaceans were cyclopoid copepods, *Bosmina*, calanoid copepods, *Chydorus*, and *Daphnia*. In this study, no correspondence was found between the Carlson indices and species richness, the equivalent number of species, or the Shannon index. Similarly, in Brazil, Attayde and Bozelli (1998) and Baião and Boavida (2005) reported no relationship between zooplankton diversity variables and trophic state, nor with the total or individual abundance of each zooplankton group. Lodi et al. (2011) also found no significant relationship between rotifer species richness and trophic state, a finding consistent with our results. In contrast, Attayde and Bozelli (1998) observed an increase in total zooplankton density with increasing trophic state.

The CCA results further support these patterns. Although El Llano was not clustered with El Cedral and Zempoala, it exhibited similar characteristics, showing negative relationships with SRP, nitrates, and chlorophyll *a*. Low-trophic-level indicators, such as Secchi disk depth, were associated with *Daphnia* spp., *Asplanchna priodonta*, and *Synchaeta* spp. In other studies, *Asplanchna priodonta* and some species of *Synchaeta*, such as *S. longipes*, *S. stylata*, and *S. tremula*, have been linked to low saprobic levels (Slácedêcek, 1983). More recently, *Synchaeta grandis* has been associated with low trophic states in central Sweden (Ejsmont-Karabin, 2012).

Eutrophication typically increases the prevalence of low-quality food, such as cyanobacteria, which reduces *Daphnia* abundance (Taipale et al., 2019). In the eutrophic systems analyzed here, high fish predation may further contribute to low *Daphnia* densities (Alcocer et al., 2022). Eutrophication has also been recognized as an important driver of the spatial distribution of the tropical genus *Diaphanosoma* (Nascimento et al., 2023), which we recorded in Xochimilco, La Estanzuela, Chapa de Mota, Chimaliapan, and Valle de Bravo. This genus swims faster than *Daphnia*, enabling it to persist in eutrophic water bodies with high predation pressure.

In temperate latitudes, the presence of brachionids, particularly species of the genera *Brachionus* and *Keratella*, has been widely recognized as indicative of mesosaprobic conditions (Slácedêcek, 1983) and eutrophication (Duggan et al., 2001a; Ejsmont-Karabin, 2012; Kuczyńska-Kippen et al., 2025). In our study, the genus *Brachionus* was present only in La Estanzuela, Xochimilco, and Chapa de Mota, which were the three aquatic systems with the highest trophic levels. In contrast, *Keratella* and most of the rotifers indicative of high trophic levels (*Brachionus angularis*, *Keratella quadrata*, *K. cochlearis tecta*, *Filinia longiseta*, and *Pompholix sulcata*) coincided with previous records for temperate lakes (Kuczyńska-Kippen et al., 2025), showing high abundances in Chimaliapan, Xochimilco, Chapa de Mota, Valle de Bravo, and La Estanzuela. Despite some similarities in indicator species, the results do not entirely correspond with TSI_SD_ and TSI_CHL_. Thus, further research should consider seasonality and include additional approaches, such as those based on biomass.

## Conclusions

This research demonstrated a clear relationship between the trophic state of lakes and reservoirs in Central Mexico and the structure of the zooplankton community, supporting the use of these organisms as reliable biological indicators of ecological status. Indices based on rotifer abundance, as proposed by Ejsmont-Karabin (2012), produced results similar to those obtained using Carlson’s TSI_SD_ and TSI_CHL_; however, indices based on microcrustacean abundances, the Ejsmont-Karabin and Karabin (2013) indices, more accurately reflected the trophic status of the water bodies.

No relationship was detected between trophic status and common diversity measures, including species richness, the effective number of species, and the Shannon index. In all water bodies, rotifers accounted for the highest number of species and the greatest abundance, except in El Cedral, El Llano, and Chimaliapan, where copepods dominated in abundance.

The low trophic level was characterized by the presence of *Asplanchna priodonta*, *Synchaeta* sp., and *Daphnia*, whereas high trophic level aquatic systems were associated with brachionids, *Filinia longiseta*, and *Diaphanosoma*. Further studies that incorporate seasonal variation are needed to improve understanding of the role of zooplankton as a biological indicator of trophic state in Central Mexico.

## Data Availability

No datasets were generated or analysed during the current study.
